# Supplementary material for: Factors associated with infants’ sunlight exposure among mothers attending the EPI unit of Wolkite University Specialized Hospital
Source: PLoS One. 2022 Nov 17;17(11):e0277349. doi: 10.1371/journal.pone.0277349 (PMC9671437; doi:10.1371/journal.pone.0277349)
Supplement: Attachment — Submitted filename: Responses to Reviewer #1.docx [file pone.0277349.s002.docx]

Responses to Editor(s) and Reviewers

Manuscript Id: PONE-D-21-36839

Title: Factors Associated with Infants’ Sunlight Exposure among Mothers Attending EPI of Wolkite University Specialized Hospital

Dear editor(s) and Reviewers I would like to say thank for your comments and recommendations. I have included the concerns raised by reviewers in the documents, and if there is untouched points I am ready to amend. Thank you again.

|  | **Reviewer #1** |  |
| --- | --- | --- |
| Section | Comments | Responses |
| Abstract part page-2 | Q1. line-26 EPI please! change to Expanded program on immunization(EPI) | Changed |
|  | Method part page-2 *please! write the abstract part properly and clearly because it is a mirror of your manuscript including sampled number of population, study design and sampling technique | Revised and the raised concerns are incorporated. |
|  | Q2. what was the study design? | Included |
|  | Q3. what was the sampling techniques? | Included |
|  | Result part page-2  Q4. line-32. how many infant coupled mothers were recruited in your study? please! start like A total of ........infant coupled mothers who visited EPI for vaccinating their children were included in the study. | The result part is revised and modified. |
|  | Conclusion part page-2 Q5. Your conclusion is more than your result and please! revise it properly | Revised and updated |
| Introduction part page-3 | Q6. line 49 UVB please! change to ultra violet B (UVB) | Changed |
|  | Q7. pge-5 line 107 EPI please! change to Expanded program on immunization(EPI) | Changed |
| Materials and methods part page 6 | Q8. inclusion and exclusion part line 128: what was the age infants which were included in the study? please! mention properly in the inclusion part. | Included |
|  | Q9. There was no any information on how authors selected 220 infant coupled mothers for this study? no sample size estimation information? is it single/two population proportion formula? purposive? | Sample size determination is included in the document. |
|  | Q10. ......knowledge, and practice questions. Every day, the filled questionnaire was checked for completeness by the principal investigator to increase data quality. How authors categorized the knowledge level practice level of of mothers? or nothing was defined about level of knowledge and practice? nothing was mentioned in the manuscript. How many Likert scaled questions were applied? Have you use principal component analysis? or threshold formula or mean/median score? | An operational definition is added in the document for both knowledge and practice questions. |
| Result | Q11. result part page 9; Please! remove ANC visit rows from table two because it doesn't give any meaning | Removed |
|  | Mothers’ knowledge about sunlight exposure of neonates part page-9  Q12. line 183-184. The overall knowledge of sunlight exposure of infants among mothers was 51.8%. How did you get this proportions? what was your analytic approach to calculate this proportion? | This is explained under the operational definition. |
|  | The practice of Sunlight Exposure part page 11.  Q13. line 197-198 The overall practice of sunlight exposure among them were 67.3% during the study period. Similar to Q12. | This is explained under operational definition. |
| Discussion part page-15. | In general the conclusion part was written poorly and the authors' requested to re-write this part by reviewing different literatures which were conducted in different parts with similar topic. | The conclusion is revised and updated. |
|  | **Reviewer #2** |  |
| Introduction: | 1. The Introduction section needs to be summed up concisely. | Rearranged |
|  | 2. The author may want to provide the prevalence of vitamin D deficiency among infants in the study area to show that sunlight exposure is very important, since “vD deficiency in order of magnitude was found in 62%, 28%, and 6% of Mexican, Cincinnati and Shanghai infants, respectively (p<0.001)”. | It is included in the document |
|  | 3. The logic flow of the introduction needs to be improved. For example, you need to provide the association between vitamin D and calcium before talking about “In African countries, calcium intake is below recommendations”. | It is added and rearranged |
|  | 4. The authors need to describe the importance/innovation of this study. Is there a knowledge gap in the research question? Is this study better than prior studies? | Included in the document, in the colored part of the literature review |
| Method: | 1. A comprehensive literature review is expected in the method section to identify the potential factors associated with sunlight exposure among infants from prior publications. | In reviewing the submission guideline, I found that a comprehensive literature review is under the introduction part. I added it at the end of the introduction part which is highlighted. |
|  | 2. The authors may want to emphasize the representative of the study population when describing the study hospital. | Even though the Hospital is a specialized hospital, it is surrounded by rural villages where it serves them in addition to referral cases. |
|  | 3. The audience would like to read about sample size and power calculation in the method section. | Included |
|  | 4. Please provide more information about the design of the questionnaire and the validation of the questionnaire. | Included |
|  | 5. Please describe the training of interviewers before the study to improve the quality of the data collection. | Included |
| Results: | 1. The authors may want to add the “outdoor” to the sunlight in the questionnaire since indoor sunlight exposure may not lead to vitamin D synthesis. | Added |
|  | 2. The distribution of “sunlight is beneficial” and “sunlight is harmful” were conflict with each other in Table 3. | Revised |
|  | 3. Please add the unit of monthly income in table 1. | Added |
|  | 4. The percentages mentioned in the Tables 3-4 were not consistent with the percentages mentioned in the result section. | Sorry, it was a mistake done during descriptive analysis. Rather than using Valid percent, we used the normal percent. Now the table and the descriptions are revised and updated. |
|  | 5.“162(73.6%) of mothers exposed their infants to sunlight. Of these,146(66.4%) started sunlight exposure from 3weeks” (row 189) was not provided in table 4. |  |
|  | 6. Please keep two decimal places for ORs. | Changed |
|  | 7. Please describe the definition of the dependent variable for the regression model. The sample size for good practice is 148 in table 5, which is not consistent with any prior tables. The 148 is consistent with figure 2, but the audience still could not find the definition of “good practice” in the manuscript. | Included |
| Discussion: | 1. In the first paragraph of the discussion, please just emphasize the most important findings. | Addressed |
|  | 2. Please add strengths, limitations, biological mechanisms, and public health implications in the discussion section. For example, please discuss the potential selection bias and limited generalizability due to the use of “specialized” hospitals and the description of the sampling method. | Revised and updated |
| Conclusion | It is too early to provide public health policy recommendations based on one cross-sectional study among participants from one specialized hospital “Government should prepare a guideline on time of exposure and duration including starting age.”, “Each health institution has to work in providing health education for mothers during ANC visits, during discharge after delivery. Health education that could be given home to home by health extension workers for rural mothers. Government should prepare a guideline on time of exposure and duration including starting age.” | Revised and updated |
| Grammar: | 1. The following sentences need to be improved for readability:   1) “The practice was determined by the age of mothers and their residence.” 2) “Compared with other previous studies used in discussion, there is progress from time to time even though the study area was not the same.” 3) 3) “Being in the age category of 30–34, 3.095 [AOR= 3.095, 95%CI (1.126, 8.506)], being in the age category of 35+ were 4.491 [AOR= 4.491, 95%CI (1.196, 16.863)], Living in urban 1.938 [AOR= 1.938, 95%CI (1.053, 3.566)] more likely to expose infants to sunlight than living in rural” 4) “Health education that could be given home to home by health extension workers for rural mothers.” | Revised and updated |
|  | 2. There are inconsistent capitalization, inappropriate use of abbreviations, and inconsistent fonts. | Revised |
|  | 3. No reference for the sentence for rows 48,53, 57, and 77 | Revised |
|  | 4. There is repetitive “of clothing” in Table 4 | Revised |
