# Supplementary material for: Factors associated with infants’ sunlight exposure among mothers attending the EPI unit of Wolkite University Specialized Hospital
Source: PLoS One. 2022 Nov 17;17(11):e0277349. doi: 10.1371/journal.pone.0277349 (PMC9671437; doi:10.1371/journal.pone.0277349)
Supplement: Attachment — Submitted filename: Abstract part.docx [file pone.0277349.s003.docx]

**Abstract part**

Line-26: This study aims to…. Please! Modify to “*This study aimed* to….” Or “*The aim of this study was*….”

Line 31-35: please! Modify to: *The collected data were entered into a computer using the software Epidata 3.1 version and exported to SPSS version 23 for statistical analysis. Descriptive and inferential analysis was conducted. Logistic regression analysis was done, and a
statistical association was declared at p-value < 5% and 95% confidence interval(CI).Then the results were presented using a frequency table, figures, and description*.

Line 38-39: *please! Modify to: Mothers practice of infant sunlight exposure was affected
39 by age of mothers 30–34 years, [AOR= 3.095, 95%CI (1.126, 8.506)], and age ≥35 years, [AOR= 4.491, 95%CI (1.196, 16.863)], and living in urban, [AOR= 1.938, 95%CI (1.053, 3.566)].*

**Materials and methods part page 6**

Line 113-115: please! Modify to: *Currently, it is providing emergency services, outpatients’ services, inpatients services, major and minor surgeries, maternal care, pediatrics services, antiretroviral therapy (ART) and Tuberculosis (TB) treatment clinics, laboratory, radiography services, and EPI services for the community.*

Sample size Determination part page 6

Line 124-128. The sample was calculated using a 95% confidence interval level, 5% margin of error, and 54.5%: based on this the calculated sample size is

(1.96)^2^x0.545x (1-0.545)/0.05^2^=381

*Please! Correct your sample size calculation. The sample size could be more than 380 as shown above based on your explanation. So how it could be reduced to 220? Please! Write clear explanation about it.*

**Factors associated with sunlight exposure of infant’s page 13**

**Line 213-214:** Mothers in the age group of *30–34 years and ≥35 were* 3.20 [COR= 3.20, 95%CI (1.18, 8.69)],

Also please! Correct sentence of line 220-221 in similar manner
