# Supplementary material for: Factors associated with infants’ sunlight exposure among mothers attending the EPI unit of Wolkite University Specialized Hospital
Source: PLoS One. 2022 Nov 17;17(11):e0277349. doi: 10.1371/journal.pone.0277349 (PMC9671437; doi:10.1371/journal.pone.0277349)
Supplement: Attachment — Submitted filename: Responses to Reviewer #2.docx [file pone.0277349.s004.docx]

Responses to Editor(s) and Reviewers’ comments

Manuscript Id: PONE-D-21-36839R1

Title: Factors Associated with Infants’ Sunlight Exposure among Mothers Attending EPI of Wolkite University Specialized Hospital

Dear editor(s) and Reviewers I would like to say thank for your comments and recommendations. I have included the concerns raised by reviewers in the documents, and if there is untouched points I am ready to amend.

Thank you for reviewing the paper.

| Section | Comments | Responses |
| --- | --- | --- |
| Journal Requirements: | Please review your reference list to ensure that it is complete and correct. | Upon reviewing the references, three of them were found incorrectely written and modified. |
|  | Reviewer #1 |  |
| Abstract part | Line-26: This study aims to…. Please! Modify to “This study aimed to….” Or “The aim of this study was….” | Modified |
|  | Line 31-35: please! Modify to: The collected data were entered into a computer using the software Epidata 3.1 version and exported to SPSS version 23 for statistical analysis. Descriptive and inferential analysis was conducted. Logistic regression analysis was done, and a statistical association was declared at p-value < 5% and 95% confidence interval(CI).Then the results were presented using a frequency table, figures, and description. | Modified |
|  | Line 38-39: please! Modify to: Mothers practice of infant sunlight exposure was affected by age of mothers 30–34 years, [AOR= 3.095, 95%CI (1.126, 8.506)], and age ≥35 years, [AOR= 4.491, 95%CI (1.196, 16.863)], and living in urban, [AOR= 1.938, 95%CI (1.053, 3.566)]. | Modified |
| Materials and methods part page 6 | Line 113-115: please! Modify to: Currently, it is providing emergency services, outpatients’ services, inpatients services, major and minor surgeries, maternal care, pediatrics services, antiretroviral therapy (ART) and Tuberculosis (TB) treatment clinics, laboratory, radiography services, and EPI services for the community. | Modified |
| Sample size Determination part page 6 | Line 124-128. The sample was calculated using a 95% confidence interval level, 5% margin of error, and 54.5%: based on this the calculated sample size is (1.96)2x0.545x (1-0.545)/0.052=381 Please! Correct your sample size calculation. The sample size could be more than 380 as shown above based on your explanation. So how it could be reduced to 220? Please! Write clear explanation about it. | We changed the methods sample size calculations based on your concern first we used epi info, and due to error in metnod of calculation we used correction formula. |
| Factors associated with sunlight exposure of infant’s page 13 | Line 213-214: Mothers in the age group of 30–34 years and ≥35 were 3.20 [COR= 3.20, 95%CI (1.18, 8.69)], Also please! Correct sentence of line 220-221 in similar manner | Modified |
|  | Reviewer #2: |  |
| Abstracts: | 1 The authors just need to keep two decimal points for the odds ratio | Corrected to two decimal place |
|  | 2 It is too early to suggest “Health professionals should provide health education for mothers at young ages and mothers from rural areas on the benefits of infants' sunlight exposure.” based on one cross-sectional study. | Modified |
| Introduction: | 1. Thank you for working on the introduction. If the authors would like to sum up the introduction section concisely, you can use the following detailed information to improve the re-submitted draft. The authors can 1)start with the outcome (prevalence of rickets in the study area) (The authors can use rows 90-92), and add information about the significance of rickets (mortality, disability, medical cost, et al) 2)state the association between rickets and vitamin D deficiency (The authors can use rows 58-61, 66-67, 78-81, 86) 3)provide the prevalence of vitamin D deficiency in study area (The authors can use rows 89-90) 4)state the sources of vitamin D in infant and the role of sunlight in vitamin D level (The authors can use rows 49-50, 62-65) 5)state sunlight would be the best solution due to the sufficient sunlight in study area, the lack of vitamin D supplements, and the common use of breastfeeding in study area (The authors can use rows 53-57, 68-76, 82, 88, 93, 103) 6)provide the prevalence of sunlight exposure in study area and factors affect the sunlight exposure in study area (The authors can use rows 51, 87, 94-97, 99-102) 7)add articles that indicate the lack of published study on practice of infant sunlight exposure and associated factors among study population 8)the aim of the study to address the knowledge gap (The authors can use rows 104-105). | We leave the introduction part as it is. The flow of ideas were started from benefits of sunlight exposure, vitamin D and sunlight exposure, deficiency of vit D, its epidemiology, relation to exposure, …  This is the reason for no rearranging the introduction.  But, we appreciate your suggestions. |
|  | Please also remove repeated information if the authors choose to rearrange the introduction section using this format. | We decided no to rearrange the part. |
|  | 2. If the authors added rows 94-98 as the knowledge gap to address previous comment #4 (there were no highlights in the introduction), then the authors need to provide why “The current study included the place of residence in addition to previously studied variables.” is the innovation/importance of the current study. Are there any articles indicating that place of residence is an important factor for sunlight exposure? The authors may need to add one sentence here. | Of course no, but we were interested to study it, from our clinical experience. |
| 3. Other suggestions about the introduction that might improve the introduction: | 1) Reference 10 may not support the statement | Removed |
|  | 2) The authors can use “children unexposed to sunlight “ to replace “unexposed children to sunlight” | Replaced |
|  | 3) Lack of reference in row 92 | Revised and added. |
| Method: | :1 The “10% non-response rate” is not consistent with the “which gives a 100% response rate” | you are right, but all distributed paper was returned from data collectors. When we check the questionnaire all were complete and decicded to include all. |
|  | 2 Please add the year to the “from 01 June to 30 July.”. | Added |
|  | 3 The power calculation needs to be improved. 1) The authors can use a fixed number of participants to calculate power, or the authors can calculate the number of participants required to reach the 80% statistical power at the significance level of 0.05. The authors can also calculate the minimal detectable odds ratios using the fixed number of participants and the 80% statistical power. 2) The information provided in the sample size determination section is not sufficient to calculate sample size or power using the Epiinfor 7 software. | From your comments we understand that we wrongly used epi info based sample size calculation. To make clarity, we decided to use correction formula since the population was small. |
| Results: | 1 “More than two-thirds (69.1%) of the mothers said that morning is a good time for infants’ sunlight exposure (Table 3).” is not consistent with the “99.1% “ reported in table 3. | Corrected |
|  | 2 The percentages for “Fear to expose baby on sunlight” in Table 4 are not consistent with the frequency. | Multiple resposnes are possible. |
| Discussion: | 1 For the first two paragraphs, please just mention the important findings. Please try not to repeat what you had in the result section. | Reviewed and modified |
|  | 2 Please revise the “The study could not support the cause and effect relationship between independent and dependent variables.” We usually would not use the terms “cause” and “effect” in the cross-sectional study. | We removed the the sentence |
|  | Reviewer #3 |  |
| Data analysis procedure | Please describe the variables included in the logistic regression analyses and define AOR. | Described, and defined |
| Results | the factors associated with sunlight exposure of infants, which were found in logistic regression, are unclear. The reader needs to know the coefficients, and you must define what COR is in the first mention. | Included |
|  | Include in all tables notes defining all the acronyms used. | Included |
|  |  |  |
