# Supplementary material for: Factors associated with infants’ sunlight exposure among mothers attending the EPI unit of Wolkite University Specialized Hospital
Source: PLoS One. 2022 Nov 17;17(11):e0277349. doi: 10.1371/journal.pone.0277349 (PMC9671437; doi:10.1371/journal.pone.0277349)
Supplement: Attachment — Submitted filename: Responses to Reviewer #3.docx [file pone.0277349.s005.docx]

Responses to Editor(s) and Reviewers’ comments

Manuscript Id: PONE-D-21-36839R2

Title: Factors Associated with Infants’ Sunlight Exposure among Mothers Attending EPI of Wolkite University Specialized Hospital

Dear editor(s) and Reviewers I would like to say thank you for your comments and recommendations. I have included the concerns raised by reviewers in the documents, and if there are untouched points I am ready to amend them.

Thank you for reviewing the paper.

| Section | Comments | Responses |
| --- | --- | --- |
| Journal Requirements: | Please review your reference list to ensure that it is complete and correct. | We reviewed and checked the references, all are correct and available online. |

Thank you
